# Metformin: A Possible Option in Cancer Chemotherapy

**DOI:** 10.1155/2020/7180923

**Published:** 2020-04-27

**Authors:** Chidiebere V. Ugwueze, Odunze J. Ogamba, Ekenechukwu E. Young, Belonwu M. Onyenekwe, Basil C. Ezeokpo

**Affiliations:** ^1^Ebonyi State University, Abakaliki, Nigeria; ^2^University of Nigeria Teaching Hospital, Enugu, Nigeria

## Abstract

Metformin has been used for a long time as an antidiabetic medication for type 2 diabetes. It is used either as a monotherapy or in combination with other antidiabetic medications. The drug came into prominence in diabetes and other conditions with cardiovascular risk after the landmark study of 1995 by the United Kingdom Prospective Diabetes Study which emphasized its importance. However, the drug has been used in experimental trials in various aspects of medicine and pharmacology such as in reproductive medicine, cancer chemotherapy, metabolic diseases, and neurodegenerative diseases. It has been in use in the treatment of polycystic ovarian disease and obesity and is being considered in type 1 diabetes. This study seeks to evaluate the relevance of metformin in cancer management. Different mechanisms have been proposed for its antitumor action which involves the following: (a) the activation of adenosine monophosphate kinase, (b) modulation of adenosine A1 receptor (ADORA), (c) reduction in insulin/insulin growth factors, and (d) the role of metformin in the inhibition of endogenous reactive oxygen species (ROS); and its resultant damage to deoxyribonucleic acid (DNA) molecule is another paramount antitumor mechanism.

## 1. Introduction

Metformin is one of the widely used oral antidiabetic medications. It belongs to the class called biguanides of which the other members have been withdrawn due to associated lactic acidosis. Metformin has been recognized as a first-line pharmacotherapy in type 2 diabetes management in most guidelines such as American Diabetes Association [1].

The origin of the drug is not well known but has been linked to a plant called *Galega officinalis* (goat's rue), which is rich in guanidine [2]. The ability of this herbal medicine to reduce blood glucose was shown in 1918. Some of its derivatives except metformin were used to treat diabetes but were withdrawn based on associated toxicities [3]. Metformin came to limelight in 1930 in the search for antimalarial medications where it was discovered to have anti-influenza and antidiabetic properties. Jean Sterne followed this discovery up, and in 1957, the use of metformin to treat diabetes was established. However, the recognition did not continue as metformin was less potent than other biguanides (phenformin and buformin) [4]. Indeed, metformin was only licensed in the USA in 1994. Despite this, metformin was the most widely prescribed oral antidiabetic drug in the USA in 2012 [5].

Metformin acts to lower blood glucose mainly by reducing hepatic gluconeogenesis, hence reducing glucose output from the liver. It is a potent glucose-lowering agent, reducing HbA1c by 10-15 mmol/mol (1.0-1.5%) [6]. The landmark study by the United Kingdom Prospective Diabetes Study (UKPDS) group in 1995 established the long-term cardiovascular benefit of metformin, giving rise to the resurgence in the relevance of metformin in diabetes management [7].

## 2. Chemical Structure of Metformin

Metformin is a white crystalline compound with a molecular formula of C_4_H_11_N_5_·HCl and a molecular weight of 165.65 g/mol [8]. Metformin hydrochloride is soluble in water but insoluble in acetone and ether. It comes in the following formulations: 500 mg, 850 mg, and 1000 mg and can be taken once daily or twice daily.

The two methyl substituents on metformin are responsible for the low lipophilicity of metformin. The low lipophilicity accounts for the slow passive diffusion across tissues. It requires a transporter SLC22A or multidrug-resistant and toxic compound extrusion type transporters to enter or exit the cells and tissues [9].

## 3. Pharmacokinetic Profile of Metformin

Metformin is denoted chemically as N,N-dimethylbiguanide. The drug is orally administered up to a maximum daily dose of 2550 mg in divided doses. It is readily absorbed and has a half-life of 1.5-3 hours for the immediate release form and 4-8 hours for the extended release type. The bioavailability is approximately 50-60% in a fasting state which is high despite the low lipophilicity [10]. This is due to the involvement of OCT3 (SLC22A3) transporter molecules [11]. Metformin is not bound to plasma proteins and, thus, has high apparent volume of distribution between 300 and 1000 L after a single dose and is not metabolized [12]. It usually takes about 1-2 days to reach the steady state. The therapeutic plasma level after oral administration of metformin is 0.465-2.5 mg/l which is adequate for diabetes control but much lower than the *in vitro* concentration required for apoptosis [13].

It is excreted through the kidneys by tubular secretion as the active drug so contraindicated in renal failure [14]. The elimination half-life is 4-8.7 hours.

## 4. Mechanisms of Action of Metformin as an Anticancer Drug

The mechanisms through which metformin achieves its anti-neoplastic effect include the following.

### 4.1. Metformin and Mammalian Target of Rapamycin Complex 1

Inhibition of cancer cell growth by suppressing mammalian target of rapamycin complex 1 (mTORC1). mTORC1 is a multiprotein complex which is composed essentially of protein kinase mTOR and scaffolding protein raptor [15]. Adenosine monophosphate protein kinase **(**AMPK) can directly phosphorylate tuberous sclerosis complex (TSC2) on S1387, thereby promoting its inhibition of mTORC1 [16]. The stimulatory effect of protein synthesis by mTOR emphasizes its role in the metabolism and proliferation of malignant cells [17, 18].

### 4.2. Activation of Adenosine Monophosphate Protein Kinase (AMPK)

Kahn et al. [19] showed that metformin exhibits its antineoplastic effect by activation of adenosine monophosphate protein kinase (AMPK). This involves direct inhibition of mTORC1 through phosphorylation of S722 and S792 on the mTOR binding raptor [20]. This is similar to the mechanism of the antidiabetic effect of metformin. The latter involves liver kinase B1- (LKB1-) dependent activation of adenosine monophosphate-activated protein kinase (AMPK) as highlighted by Shaw et al. [21].

### 4.3. Metformin and Inhibition of Generation of Reactive Oxygen Species (ROS)

The ROS signaling pathways are markedly increased in many types of cancers where they give rise to abnormal proliferation and differentiation. Reactive oxygen species include peroxides, superoxides, hydroxyl radicals, singlet oxygen, and alpha oxygen [22]. The role of hydrogen peroxide, a typical example of ROS, is implicated in reversible oxidation of tyrosine phosphatases, tyrosine kinases, and transcription factors [23, 24].

The inhibition of ROS generation is mediated by the action of metformin on complex 1 of the respiratory chain which reduces entry of electron to the chain and eventually ROS production [25, 26]. The concentrations of metformin required to directly inhibit complex 1 molecule is high (20-100 mM) in isolated mitochondria *in vitro* [26]. However, the *in vivo* inhibition of complex 1 molecule can be achieved with micromolar concentrations [27]. The explanation is based on the positive charge of metformin which allows slow accumulation within the mitochondrial matrix [28]. The inhibition of endogenous generation of ROS is independent of the AMPK*α* system [25]. One of the vital targets of ROS-induced cellular damage is the DNA with a consequent structural distortion of its integrity (mutation). Flow cytometry shows that cells pretreated with metformin can reduce ROS levels following paraquat exposure [29].

### 4.4. Reduction of Serum Levels of Insulin, IGF-1, and IGF-2

Metformin reduces the levels of stimuli that promote cancer cell proliferation [30]. High levels of IGF-1 and IGF-2 are linked to the growth of cancer or with cancer recurrence in cancer survivors [31]. The actions of IGF proteins are mediated by IGF-IR, a transmembrane tyrosine kinase which is structurally related to insulin receptor. The binding of IGF-1 and IGF-2 on IGF-receptors eventually results in the activation of mTOR which enhances cellular proliferation and inhibition of apoptosis [32].

### 4.5. Inhibition of Chronic Inflammation

Metformin also inhibits chronic inflammation, a process which is an important mechanism in the initiation and promotion of carcinogenesis [33, 34]. Metformin inhibits the initial activation of inflammatory response associated with cellular transformation and cancer stem cell growth. This is related to the inhibition of inflammatory transcription factor, NF-Kb [35].

### 4.6. Modulation of Adenosine A1 Receptor (ADORA1) Expression

Lan et al. [36] described a new pathway involved in the antineoplastic effect of metformin which involves the modulation of adenosine A1 receptor (ADORA1) expression in human colorectal cancer and breast cancer cells. ADORA1 receptors play essential in the supply of cellular energy. Malignant cells are, therefore, deprived of energy in the course of downregulation of ADORA1 receptors. Metformin treatment appreciably upregulates ADORA1 expression in colorectal cancer cells [37]. The ADORA1-mediated growth inhibition and apoptosis induced by metformin are AMPK-mTOR pathway dependent in human colorectal cancer cells [36].

### 4.7. Downregulation of Gluconeogenesis in the Mitochondria

The antitumor activity of metformin is related to the downregulation of gluconeogenesis in the mitochondria [21]. Hyperglycaemia modulates various pathways that control cell proliferation, migration, and invasion [38]. Warburg phenomenon refers to rapid glucose uptake and metabolism by cancer cells via a process of aerobic glycolysis for the purpose of generating energy [39]. Therefore, hyperglycaemia provides the necessary adenosine triphosphate (ATP) which is required by cancer cells to proliferate rapidly [38].

**Figure 1:**
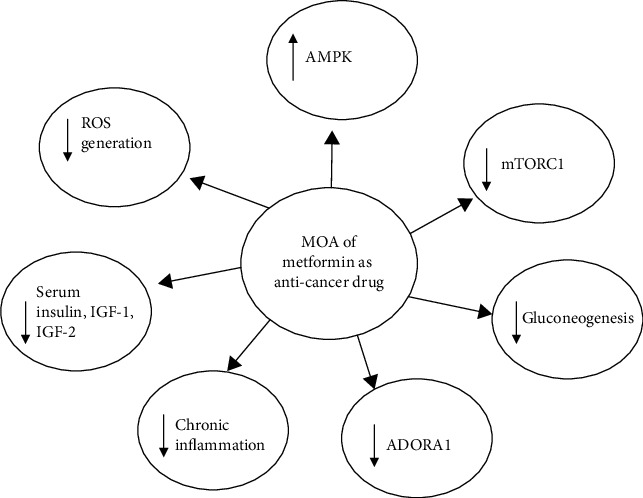
Mechanisms of antineoplastic action of metformin. MOA: mechanism of action; ADORA1: adenosine A1 receptor; AMPK: adenosine monophosphate kinase: ROS: reactive oxygen species; mTORC1: mammalian target of rapamycin complex 1; IGF: insulin-like growth factor; ↓: inhibition; ↑: activation.

The diagrammatic representation of the mechanisms of action of metformin as an anticancer agent is shown in Figure 1.

## 5. Cellular Transporters of Metformin

Several transporter proteins are involved in conveying metformin to its intracellular target of action. These transporters, which are usually polyspecific organic cations, are important due to the hydrophilicity of metformin. Transporters are involved in metformin absorption, distribution, and elimination. Several transporters have been described which include OCT1 (SLC22A1), OCT2 (SLC22A2), OCT3 (SLC22A3), MATE1 (SLC47A1), MATE2 (SLC47A2), PMAT (SLC29A4), and OCTN1 (SLC22A4) [40, 41]. Recently, thiamine transporter 2 (THTR-2) (SLC19A3) has been described to play a role in the intestinal absorption and renal reabsorption of metformin.

The cation plasma membrane monoamine transporter (PMAT; SLC29A4) has been implicated in the intestinal absorption of metformin. Others include transporters in the SLC22 family. These include OCT1 (SLC22A1) and OCT3 (SLC22A3), which are expressed in the small intestine. The transporter OCT3 is located at the brush border of the enterocytes, while OCT3 is at the basolateral membrane. Metformin has a large volume of distribution, suggesting a robust tissue uptake. This is facilitated by the transporters OCT3 and OCT1. The pharmacologic actions of metformin are attributed to OCT1 and OCT3.

Organic cation transporter 3 (OCT3) is predominant in human breast cancers. The study done by Cai et al. [42] showed that human breast cancer cell lines which were engineered to express OCT3 achieved >13 times metformin uptake and >4 times antiproliferative effects compared to the control group without OCT3. There was also increased AMPK phosphorylation in the test group.

## 6. Clinical Trials Using Metformin Hydrochloride in Cancer Therapy

Some clinical trials with metformin are ongoing to ascertain its effect in the treatment of different malignancies. However, some trials have been concluded. The trials involve metformin monotherapy or combination therapy with other chemotherapeutic agents. These are shown in Table 1.

## 7. Outcome of Some Concluded Clinical Trials

- NCT02145559: tried to determine the effect of sirolimus and metformin on solid tumors. The combination was well tolerated, but there were no significant changes in phospho-p70S6K and other pharmacodynamic biomarkers [51].
- NCT01926769: studied the effect of FOXFOX6 and metformin/FOLFIRI and metformin on colorectal cancer. The outcome is not yet available.
- NCT00930579: evaluated the effect of metformin monotherapy on the AMPK/mTOR pathway in breast tumor. There was no significant variation noted in changes of in Ki67 when metformin arm was compared to control arm (*p* = 0.47) [52]

### 7.1. Large Population-Based Studies of Metformin on Specific Cancers

#### 7.1.1. Metformin and Prostate Cancer

Prostate cancer is one of the commonest cancers diagnosed in men. A study demonstrated that out of 87,344 men with 17% of whom were diabetic and on metformin, 22% also had DM but were not on metformin; there was an 18% significant reduction in death risk as well as skeletal related events [53].

The relationship between oxidative stress and prostate cancer has been demonstrated in various clinical studies [54, 55]. The protective effect of some antioxidants in men with cancer of the prostate has also been reported [56]. However, as demonstrated in the SELECT trial and Physicians' Health Study II, some antioxidants such as vitamin E have not shown any protective role against prostate cancers [57, 58].

Studies concerning the use of metformin and the risk of developing prostate carcinoma appear conflicting. While some studies showed that metformin has the ability to reduce the risk of developing prostate cancer, others showed positive association of prostate cancer with metformin administration [59–62] However, in a cohort study by Mergel et al. [63] among 3837 patients, it was noted that the longer duration of metformin treatment after diagnosis of prostate cancer was associated with decline in all-cause mortality.

Xie et al. [64] demonstrated that metformin decreases androgen receptor (AR) and androgen receptor-V7 expression and enhances apoptotic cell death. Based on this property of metformin, it improves the antiprostate activity of abiraterone and enzalutamide combination.

Another study by Kuo et al. [65] showed a significant reduction in prostate cancer risk among 2906 metformin cohort and 2906 nonmetformin cohort who were followed up for 5-10 years (95% CI = 0.49, *p* = 0.0039).

#### 7.1.2. Liver Cancer

The risk of liver cancer was significantly reduced in type 2 diabetes mellitus patients on metformin compared to those who did not receive metformin. Bhalla et al. [66] showed there was minimal liver tumor activity in mice taking metformin compared to the control group where the tumor growth was significant. One of the proposed mechanisms is the inhibition of lipid synthetic capacity of the liver as lipids promote tumorigenesis. In a meta-analysis by Zhang et al. [67], metformin was associated with 62% reduction in the risk of liver cancer among patients with type 2 diabetes (*p* < 0.001, OR = 0.38, 95% CI = 0.24-0.59). The risk of hepatocellular cancer was even much more reduced in patients on metformin. Another meta-analysis of 19 studies by Shujuan et al. [68] which involved 550,882 diabetic subjects showed a reduction of liver cancer among metformin users by 48% (OR = 0.52; 95% CI = 0.40-0.68) compared to nonmetformin users. Among 42,217 metformin users, there was a strong inverse association between metformin and liver cancers but was not established for bladder, breast, renal, or pancreatic cancers [69].

#### 7.1.3. Metformin and Colon Cancer

Colon cancer is one of the common cancers in man. Many studies on the effect of metformin on colorectal cancer have been carried out in patients with diabetes. The prognosis of colorectal cancer in diabetic patients tended to be worse [70].

Meng et al. [71] in meta-analysis involving seven cohort studies showed that metformin use in colorectal cancer patients with diabetes improved overall survival, reduced cancer-specific mortality, but was not statistically significant (pooled hazard ratio = 0.75, 95% CI = 0.65-0.87). Mei et al. [72] in a meta-analysis of 6 cohort studies demonstrated that the metformin group had a better overall survival (HR = 0.56, 95% CI = 0.41-0.77) than nonusers. However, this meta-analysis had some limitations such as nonspecification of duration of intake of metformin and the impact of other nondiabetic medications in which participants were taking. The latter, such as additional use of insulin, could present a potent confounder.

Higurashi et al. [73] demonstrated in a randomized control trial that metformin has a chemopreventive effect on sporadic colorectal cancer in patients with high risk of recurrent adenoma. Park et al. [74] showed lower mortality in diabetic patients with colon cancer who were taking metformin than in nonmetformin users [72 (38.9%) versus 107 (46.9%), *p* = 0.012]. This study further demonstrated that female patients with colon cancer on metformin had lower specific mortality rate than their male counterparts (*p* = 0.025).

AMPK activation and inhibition of mTOR are the potential mechanisms for the anticolon cancer effect of metformin. However, the role of estrogen receptor ER*β* protein expression has been suggested and higher ER*β* protein expression was associated with higher survival rate in females than in males [75].

A prospective study which involved 47,351 participants in Northern California with diabetes but not on metformin followed up for 15 years revealed that long-term use (≥5year) was associated with reduced risk of colon cancer in the study population (HR, 0.78; 95% CI, 0.60-1.02) [76]. Further deductions from the study showed that higher cumulative doses of metformin were associated with reduced colorectal cancer risk and switching patients from sulphonylureas to metformin or adding metformin is associated with decreased colon CA risk. Similar findings have been shown by Smiechowski et al. [77].

However, the Rosiglitazone Evaluated for Cardiovascular Outcomes and Regulation of Glycemia in Diabetes (RECORD) trial was not able to demonstrate any significant cancer protective effect of metformin [78].

#### 7.1.4. Metformin and Pancreatic Cancer

Pancreatic cancer mainly refers to cancer affecting the exocrine portion of the pancreas [79]. It is the fourth most common cause of cancer death worldwide. The risk of pancreatic cancer is reduced in diabetic patients on treatment with metformin as shown by Andriulli et al. [80] A meta-analysis of 8 studies which involved 4293 patients with pancreatic cancer coexisting with diabetes showed that among the 2033 patients on metformin, there was a relative survival benefit (hazard ratio, 0.81; 95% confidence interval, 0.70-0.93) [81]. The meta-analysis established that among the study population of 4293 diabetic patients with pancreatic cancer on metformin achieved 19% survival benefit compared to nonmetformin users. However, other researchers have shown contradictory result (Hwang et al. [82] and Reni et al. [83]).

#### 7.1.5. Metformin and Breast Cancer

Some studies have reported decreased breast cancer incidence and/or mortality in patients with diabetes who are receiving metformin relative to those receiving other antidiabetic drugs [84, 85]. Breast cancer like other cancers arises when cells lose the ability to halt the process of mitosis, in addition to resistance to apoptotic cell death. High levels of phosphatidylinositol-3-kinase (PI3K)/Akt and mammalian target of rapamycin (mTOR) signaling molecules are expressed by breast cancer cells which impair their ability to undergo apoptosis [86].

Metformin inhibits the inflammatory pathways which are induced by hyperglycaemia and insulin resistance. This indirectly acts by deactivating AMPK pathways, thereby allowing the anticancer effects of metformin to be exhibited [21]. Metformin also works synergistically with chemotherapeutic agents and reduces the development of resistance of breast cancer to them [87].

The mechanism of metformin action in breast cancer is not limited to AMPK pathways. Metformin also induces cell cycle arrest, giving rise to sub-G_1_ populations and activating apoptotic pathways through downregulation of differentiated embryo chondrocyte 1 (DEC1) and p53 [88]. Administration of metformin has also been shown to cause an increase in intracellular ROS by disruption of the mitochondrial electron transport chain and reduction in the mitochondrial membrane potential [89]. Metformin exhibits proapoptotic effects and promotes cell cycle arrest via increased oxidative stress, as well as AMPK and FOXO3a activation [88].

A retrospective study carried out among Taiwanese women with type 2 DM showed that among 285,087 nonmetformin users, 9322 (2.10%) developed breast cancer (Ca) while among 191,195 metformin users, 2412 participants (1.26%) had breast cancer [90]. Thus, metformin use is associated with a decreased risk of breast cancer. Another retrospective study by Aksoy et al. [91] showed that among 784 breast cancer patients, metformin users had better clinicopathological properties compared to metformin users (*p* = 0.03).

Implicated in the molecular mechanism of metformin in breast CA is the inhibition of STAT3 phosphorylation in triple-negative and HER2-positive breast cancer [92]. The effect is that of inhibition of cellular proliferation and induction of apoptosis. This is mediated by reduction in the phosphorylation of Tyr 705 and Ser727 [93].

The antineoplastic action of metformin has also been related to its ability to increase miR-26b and a reduction in visfatin levels [94]. Sheikhpou [95] postulated that an increase in visfatin level is linked to malignant characteristic and adverse prognosis in breast cancer. This is mediated through c-Abl and STAT3 oncoproteins.

## 8. Metformin as Adjuvant to Standard Chemotherapy/Radiotherapy

Tumor cells often times develop multidrug resistance to many chemotherapeutic agents in use. Conversely, metformin may prevent multidrug resistance in breast cancer [96]. Metformin may also resensitize cancer cells to some standard chemotherapeutic agents which they were initially sensitive to. Liu et al. [96] and Qu et al. [97] demonstrated that metformin alters the features of multidrug resistance and resensitizes cells to 5-fluorouracil, adriamycin, and paclitaxel through AMPK and mTOR pathways in breast cancer.

Metformin also blocks the pathways for nicotinamide adenine dinucleotide (NAD^+^) regeneration [87]. Cell death eventually results in the depletion of NAD^+^.

Metformin also improves the sensitivity of cancer cells to radiation therapy [98]. The drug may cause superoxide generation by the activation of pyruvate dehydrogenase and *α*-ketoglutarate dehydrogenase complexes through NADH-dependent mechanism [89].. ROS causes structural damage to DNA of the tumor cells. Samsuri et al. [99] showed that patients with squamous cancer of the oesophagus who received radiotherapy and metformin had improved outcomes.

Metformin is also in phase I/II clinical trial with cisplatin and external beam radiation therapy in participants with stage III-IV head and neck squamous cell cancer [100]. This is actively ongoing in 3 locations at the United States of America. Metformin hydrochloride functions by blocking some enzymes required for cell growth. Cisplatin works by either killing the cells or stopping them from dividing by forming DNA adducts and preventing DNA repairs [101]. External beam radiation kills tumor cells and shrinks tumors.

## 9. Limitations/Challenges

1. The dose of metformin used *in vitro* is very high, usually between 10 and 40 mM (1650-6600 mg/l) and up to 100 mM in isolated mitochondria which is much greater than the therapeutic plasma levels (0.465-2.5 mg/l) in humans [13, 102]. Therefore, the challenge is how to achieve such therapeutic dose in the serum without toxic effects. However, a recent study has shown that concentration of 10 mmol/l can be used in vitro to inhibit cancer proliferation but higher concentrations may be required to achieve apoptosis [103]. The cell culture media used to achieve optimal condition for tumor cells contain high concentrations of growth factors, hormones, and glucose which are nonphysiological. Dowling et al. [104] attributed the elevated metformin dose required *in vitro* to high concentrations of the culture media constituents
2. The *in vitro* studies were conducted outside the confines of the human body; therefore, the notable effects of metformin may not have relevance in clinical settings. The focus should be on how to obtain culture media with physiological concentration of growth factors and glucose, so that the effects of metformin noted on isolated tumor cells can be extrapolated to humans [104]

## 10. Is Metformin Carcinogenic?

Metformin contains a compound called N-nitrosodimethylamine (NDMA). At an average daily intake of greater than 96 nanograms, NDMA possesses carcinogenic property [105]. This led to a recent evaluation of NDMA levels in different versions of metformin by the Food and Drug Administration (FDA). However, the findings from laboratory evaluation showed that FDA-approved metformin products do not contain dangerous levels of NDMA [106].

## 11. Conclusion

Metformin is well-known for its use in the treatment of patients with diabetes mellitus. Several molecular properties such as the inhibition of reactive oxygen species, mTORC1. ADORA and activation of AMPK have suggested its utility as an antitumor agent. A few clinical trials have been conducted to investigate its use in some tumors, but results have not been encouraging, though other trials are still ongoing. Several population studies have suggested a protective effect of metformin in the cancer of the breast, colon, pancreas, prostate, and liver. However, these have not clearly demonstrated a role for metformin as either a chemotherapeutic agent or adjuvant therapy. As the results of ongoing clinical trials are awaited, the authors suggest that further investigational research may focus on validating these findings with the aim of metformin use in cancer chemotherapy even in nondiabetic patients also. Furthermore, correlating the metformin dose used in clinical medicine with the concentration used *in vitro* may enhance the clinical utility of metformin in neoplasms.

## Figures and Tables

**Table 1:** Clinical trials with metformin and other chemotherapeutic agents.

Cancer type	Phase	Primary outcome	Dosing regimen	Combination	Enrollment number	Clinical trial ref	Completion time
Breast cancer [43]	0	Changes in Ki67	Metformin+atorvastatin	Metformin, atorvastatin	40	NCT01980823	2021
Breast cancer [44]	II	Effects of metformin on AMPK/m/TOR pathway	Metformin 1500 mg daily for 2 weeks before surgery	Metformin monotherapy	35	NCT00930579	2014
Breast, lung, liver, kidney [45]	I	Effect of metformin+sirolimus on p70S6K	Sirolimus 3 mg daily for 1-7 days. Metformin 500 mg once daily for 8-21 days	Metformin, sirolimus	64	NCT02145559	2018
Colorectal [46]	II	Disease control rate	FOLFOX+metformin/FOLFIRI+metformin	Metformin, FOLFOX6	48	NCT01926769	2014
Prostate [47]	I	DLT assessed at 28days	Enzalutamide PO QD and metformin PO BID	Metformin, enzalutamide	24	NCT02339168	2021
Pancreas [48]	II	PFS at 12 months	Everolimus+octreotide LAR+metformin	Metformin everolimus, octreotide LAR	43	NCT02294006	2017
Chronic lymphocytic leukemia [49]	II	Time to treatment failure (assessed 3 months)	Metformin 500 mg PO QD, increased to 500 mg BID after 1week and to 1000 mg BID at week 3	Metformin monotherapy	53	NCT01750567	2020
Breast cancer, early stage [50]	III	DFS for a 10-year duration	Arm I: oral metformin HCl QD for 1-4 weeks, then BID afterwards. Treatment for 5 years Arm II: placebo given the same way as metformin in Arm I.		3649	NCT01101438	2022

PFS: progression-free survival; RFS: relapse-free survival; DLT: dose-limiting toxicity; FOLFOX: folinic acid, fluorouracil, oxaliplatin; QD: once daily; BID: two times daily; FOLFIRI: irinotecan, folinic acid, fluorouracil.
